# The Impact of Adenosine Fast Induction of Myocardial Arrest during CABG on Myocardial Expression of Apoptosis-Regulating Genes Bax and Bcl-2

**DOI:** 10.4061/2009/658965

**Published:** 2009-12-22

**Authors:** Ahmed Shalaby, Timo Rinne, Otso Järvinen, Juha Latva-Hirvelä, Kristiina Nuutila, Antti Saraste, Jari Laurikka, Helena Porkkala, Pekka Saukko, Matti Tarkka

**Affiliations:** ^1^Division of Cardiothoracic Surgery, Heart Center, Pirkanmaa Hospital District, P.O. Box 2000, 33521 Tampere, Finland; ^2^Department of Forensic Medicine, Central Hospital, Turku University, Kiinamyllynkatu 6-8, 20520 Turku, Finland

## Abstract

*Background*. We studied the effect of fast induction of cardiac arrest with denosine on myocardial bax and bcl-2 expression. *Methods and Results*. 40 elective CABG patients were allocated into two groups. The adenosine group (*n* = 20) received 250 *μ*g/kg adenosine into the aortic root followed by blood potassium cardioplegia. The control group received potassium cardioplegia in blood. Bcl-2 and bax were measured. Bax was reduced in the postoperative biopsies (1.38 versus 0.47, *P* = .002) in the control group. Bcl-2 showed a reducing tendency (0.14 versus 0.085, *P* = .07). After the adenosine treatment, the expression of both bax (0.52 versus 0.59, *P* = .4) and bcl-2 (0.104 versus 0.107, *P* = .4) remained unaltered after the operation. *Conclusion*. Open heart surgery is associated with rapid reduction in the expression of apoptosis regulating genes bax and bcl-2. Fast Adenosine induction abolished changes in their expression.

## 1. Introduction

Apoptosis has been considered as one of the mechanisms of cell loss during ischemia/reperfusion (I/R) injury [1–3]. The presence of cardiomyocyte apoptosis (CA) is evident early after cardioplegic arrest and open heart surgery both in animal models and humans [4, 5]. The mechanism underlying I/R-induced apoptosis as well as its clinical significance during open heart surgery remains incompletely elucidated [4]. However, recent studies demonstrate that cardioplegic arrest immediately activates the myocardial apoptosis signal pathway. 

Myocardial upregulation of the proapoptotic gene bax and reduction of both bcl-2 and bcl-2/bax ratios are predisposing the cardiomyocytes to apoptosis [6, 7]. Their changes in response to transient ischemia have been described in experimental models and in the border zones of human myocardial infarction. Furthermore, inhibition of apoptosis during reperfusion is associated with reduction in infarction size, improvement in regional contractile and vascular endothelial functions, as well as augmentation in myocardial blood flow [8–14].

Injection of adenosine into the aortic root followed by blood cardioplegia solution after cross-clamping, produces significantly faster cardiac standstill in patients with severe coronary artery disease [15, 16]. Adenosine attenuates postcardioplegic dysfunction in severely injured hearts through the operation of receptor-mediated mechanisms [17–20]. In an experimental animal model, adenosine inhibits apoptosis via modulation of antiapoptotic bcl-2 and proapoptotic bax genes and neutrophil accumulation, primarily mediated by an adenosine A2a receptor [21–23].

The main motive behind our present study is based on the previous findings [22, 23] representing the fact that apoptosis represents a potentially preventable form of cell death owing to its active nature, specially that these findings may have important clinical implications as new cardioprotective treatment strategies are developed.

The aim of this study was to determine whether cardiac surgery using cardiopulmonary bypass (CPB) is associated with changes in the expression of apoptosis-regulating genes bax and bcl-2 in humans. We also studied whether more rapid induction of myocardial arrest by a single bolus injection of adenosine followed by potassium blood cardioplegia modifies their expression. We hypothesized that analysis of bax and bcl-2 changes in genes expression might serve a marker of myocardial injury and success of cardioprotection early after cardiac surgery with cardioplegic arrest.

## 2. Material and Methods

### 2.1. Patient Selection

The main cohort of this study included 40 patients who were scheduled for an elective CABG procedure using on-pump CPB technique. The exclusion criteria were diabetic patients with sulfonylurea medication, unstable angina, recent myocardial infarction within the last month, redo cardiac operation, preoperative diagnosis of asthma, chronic obstructive pulmonary disease; the patients did not have a history of chronic renal or hepatic diseases, and their values of creatinine (50–90 female, 60–100 male, 115 mmol/L) and aP-TT-INR (International normalised ratio; 0.9–1.2 ) were within normal range. Patients with poor left ventricular function (ejection fraction EF ≤ 30), valvular disease, and those receiving corticosteroids were also considered not eligible. After institutional approval by the Ethics Committee of Tampere University Hospital, the protocol for this prospective randomized, double blind, placebo-controlled study was reviewed by National Agency for Medicines, Finland. All 40 patients gave their informed consent.

In our previous publication [24] we had collected the data from the same study cohort but in the present report we have added more end points based on more comprehensive analysis in order to better understand the mechanisms of cardioplegic arrest.

### 2.2. Anaesthesia

A radial artery line and a pulmonary artery catheter were inserted for haemodynamic monitoring. Anaesthesia was induced with propofol (0.5–1.0 mg/kg), sufentanil (0.6–0.8 *μ*g/kg), and cisatracurium. Sufentanil infusion was continued with a rate of 0.03–0.05 *μ*g/kg/min. Sevoflurane was used as the main anaesthetic agent throughout the operation, and also provided during the cardiopulmonary perfusion with a vaporiser attached to the fresh gas inlet.

### 2.3. Operative and Perfusion Techniques

The surgical techniques were standardized in all cases, including median sternotomy, and one internal thoracic artery, along with one to four peripheral veins from the lower extremities harvested in each case. Left radial artery was harvested whenever indicated. CPB was established with standard cannulation technique, using mild hypothermia (35°C) with nonpulsative flow with a membrane oxygenator. The circuit was primed with 1500 mL of Ringer's acetate. The proximal anastomoses were constructed during a single cross-clamping period. 

### 2.4. Cardioplegia and Adenosine Administration

Patients randomization was between January 2006 and May 2007. Patients were allocated into two groups. In the adenosine group, 20 patients received 250 *μ*g/kg adenosine into the aortic root just after cross-clamping. This dose was chosen in view of a pilot study to be the lowest effective dose to stop the myocardium. The plan is to use Adenosine as a single bolus dose to allow the evaluation of adenosine not only as a pretreatment during the reperfusion but also as a maintenance dose of myocardial protection during ischemia and reperfusion.

20 patients in the control group received normal saline as placebo. All patients received standard blood cardioplegia delivered through antegrade route. The basal CP concentrations were identical in both groups, except for the study medication. Concentrations of potassium and magnesium depend on the mixing ratio of cardioplegia rollers. Ratio 1 : 4 is used for the induction, 1 : 8 for the subsequent infusions and also for the warm terminal infusion.

Total volume of cardioplegia (CP) was not recorded. Cardioplegia was administered by means of time, pressure, and flow (according to the guidelines by Dr. Buckberg). CP was infused via antegrade route with pressure of 60–80 mmHg. Maximal flow was 300 mL/min. Duration of the induction infusion was three minutes. Additional infusions after each distal anastomosis were for one minute, and the final warm infusion was given for three minutes. The effect of adenosine is known to be dependent on the temperature [2]. Therefore, the first cardioplegia infusion was given at normothermia (36-37°C) until asystole of the heart was acquired, or at least for one minute if asystole was acquired sooner. Thereafter, the cooling device setting was 10°C which yielded CP of 12-13°C. Subsequent one-minute antegrade cardioplegia infusions were administered after completion of each distal anastomosis. Final warm CP was given at normothermia; that is, 36-37°C.

### 2.5. Tissue Harvesting

From each patient, we harvested two samples from the right atrium at two different times. The first one was obtained before cross-clamping as the baseline biopsy and the second from the same location just before CPB was discontinued. Myocardial samples (5–10 × 3 mm) were immediately frozen in liquid nitrogen for assessment of gene expression.

Two samples of left ventricular apex were harvested in both groups. The first sample was taken immediately after CPB was established by oblique introduction of Tru-Cut needle (PRECISA TM 14G X 150 mm) into the left ventricle apical wall. The second sample was taken from the same location by the same needle before weaning from CPB. The puncture sites were secured with small 4-0 prolene (Ethicon^®^) stitches even when no bleeding occurred. Myocardial tissue (5–10 × 3 mm) was immediately frozen in liquid nitrogen for histological studies.

### 2.6. Total RNA Extraction and Complementary DNA Synthesis

Atria samples were homogenized and total RNA was extracted with Trizol reagent (Invitrogen^®^) according to manufacturer's instructions. The RNA concentration was determined using spectrophotometry. Total RNA (1 *μ*g) from each sample was incubated with equal amount of random hexamers (Promega*™*) at 70°C for 10 minutes and then cooled on ice for 5 minutes. Complementary DNA (cDNA) was synthesized in a mixture containing M-MLV Reverse Transcriptase RNase H Minus-enzyme (200 U), Recombinant RNasin Ribonuclease Inhibitor (40 U), dNTP mix (25 mM each), M-MLV RT 5x buffer, and Nuclease-free water (all purchased from Promega Corp. WI, USA). Mixtures were incubated at room temperature for the initial 10 minutes, then at 50°C for the final 50 minutes. Finally, the reaction was inactivated by heating for 5 minutes at 95°C.

### 2.7. Primers and Probes

Sequences of the oligonucleotide primers and probes used in real-time quantitative reverse transcriptase polymerase chain reaction (RT-PCR) assay were the same that used for bax and bcl-2 [25] (Sequence detection primer, Applied Biosystems Inc, CA, USA) and probes (TaqMan^®^ TAMRA*™* Probe, Applied Biosystems) used in real-time quantitative reverse transcriptase polymerase chain reaction (RT-PCR) assay. In order to prevent the amplification of contaminating genomic DNA, primer pairs were located in different exons. Housekeeping gene 18S rRNA was used as an endogenous control (The TaqMan^®^ Ribosomal RNA Control Reagents, Applied Biosystems).

### 2.8. RT-PCR Assay

The quantitative real-time RT-PCR was performed using Applied Biosystems 7500 RT-PCR system and Taqman^®^-based chemistry. The PCR solution-composed of 5 ng cDNA, forward and reverse primers (400 nM), specific probe (100 nM), and TaqMan^®^ Universal PCR Master Mix. Primer and probe concentrations for 18S were 50 nM and 200 nM, respectively. After incubation at 50°C for 2 minutes and denaturing at 95°C for 10 minutes, PCR was carried out, 50 cycles of 95°C for 15 seconds and 60°C for 1 minute. All RT-PCR reactions were run as a duplicate.

### 2.9. Quantification of Gene Expression

Quantification of gene expression was carried out by using relative standard curve method. Briefly, standard curves for each plate were generated using serial dilutions of cDNA synthesized from human tonsil tissue RNA. The amounts of both target and endogenous control cDNA in each sample were calculated relative to the standards. To eliminate the variation of total cDNA quantity in each sample, the results are expressed as a ratio of the target gene to the endogenous control.

### 2.10. Assessment of Apoptosis in Left Ventricular Tissue

Apoptosis was detected using the terminal transferase mediated ddUTP nick end-labeling (TUNEL) assay, as previously described [26, 27]. In brief, paraffin-embedded myocardial sections were heated in sodium citrate solution and digested with proteinase-K to expose DNA. The DNA strand breaks were then labeled using terminal transferase with digoxigenin-conjugated ddUTP and visualized using alkaline phosphatase immunohistochemistry (IHC). To confirm optimal sensitivity of the assay, it was standardized with the use of serial sections treated with DNase I to induce enzymatic DNA fragmentation (positive control of apoptosis). The apoptotic rate was defined as the positive TUNEL cardiomyocytes per field, and expressed as % of cardiomyocytes.

### 2.11. Statistical Analyses

Statistical analysis was performed using SPSS for Windows software, version 9.0 (SPSS; Chicago IL, USA). The Mann-Whitney *U* Test was used to distinguish demographic differences in means between the groups. Continuous variables were analyzed by analysis of variance (ANOVA) for repeated measures. Logarithmic transformation, median and quarter analysis methods were used when the variables were not normally distributed. Statistical significance was attributed to *P* value <.05.

## 3. Results

### 3.1. Patient Characteristics

**Demographic and Basic Clinical Data.**

The study cohort included 40 patients between the ages of 46 and 73 years, 90% of them were male patients (Table 1). Twenty patients were given adenosine and the others served as controls. All the patients in both groups completed the study protocol. There were no adverse effects related to adenosine. There were no adverse postoperative complications in any of the 40 patients. All the patients survived the operation and were discharged from the hospital. There were no adverse effects related to adenosine. There was no significant difference in the baseline characteristics of the patients between study groups as shown in Table 1.

**Figure 1:**
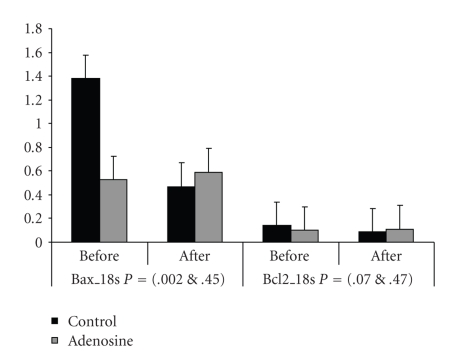
Baseline versus the postoperative expressions of bax and bcl2 in adenosine treated and the control groups. Gene expression is normalized to the same endogenous gene. Before and After refer to the timing of taking the biopsies (Before; before cross clamp application and After; after cross clamp removal and just before CPB discontinuation).

As shown in Table 2 and Figure 1, in the control group, expression of bax gene was significantly reduced in the postoperative samples as compared with the preoperative samples (*P* = .002). Expression of bcl-2 showed a similar reduction tendency (*P* = .07). However, in contrast to the control group, expression of both bax and bcl-2 remained unaltered before and after the operation in the adenosine treatment group (*P* = .45 and .47). There were no significant differences in the level of bax/bcl-2 ratio expression in either pre- or postoperative samples in the control and adenosine groups.

As shown in Table 2, (the ventricular samples were used to detect the apoptotic index and then patients were allocated into subgroups accordingly to TUNEL positive and negative, meanwhile the atria samples were used to detect the gene expression of apoptosis) there were no significant differences in either expression or bax/bcl-2 ratio between patients who had or had not TUNEL positive cardiomyocytes in the left ventricular tissue samples. Neither was there correlation between gene expression nor the amount of TUNEL positive myocytes. However, there was a tendency towards lower bcl-2 expression and higher bax expression in patients with TUNEL positive myocytes as shown in Table 2.

Values are presented as median values and standard deviation. “Before” refers to before cross-clamp application and “After” refers to after cross-clamp removal and just before CPB discontinuation.

## 4. Discussion

We found a decrease in apoptosis regulating genes in right atria samples obtained immediately after reperfusion compared to preischemic samples in patients undergoing CABG. Interestingly, expression remained stable in patients randomized to adenosine treatment during cardioplegia. Also, in the adenosine group, the bax/bcl-2 ratio decreased more than in the control group, although the decrease was not statistically significant. 

Previously, in a canine model, adenosine reduced apoptosis induced by I/R injury. This reduction was associated with enhanced expression of antiapoptotic bcl2 genes. Adenosine also reduced expression of proapoptotic bax gene in the peri-infarct myocardium [21]. Our results are consistent with the previous in that the level of antiapoptotic bcl-2 expression decreased after ischemia in the control group while the adenosine treatment prevented this down-regulation [21]. In contrast to the previous study, we found that also the expression of proapoptotic bax was reduced in the control group while its expression remained stable with adenosine treatment. This may be related to very short time reperfusion (versus 6 hours of reperfusion in the earlier study). Cardioplegic protection during ischemia may suppress proapoptotic signaling, and RNA expression may not directly reflect protein levels that were measured in the earlier study. 

In parallel with the observed changes in the expression of apoptosis-regulated genes, the amount of apoptotic cardiomyocytes as detected with the TUNEL-assay in the left ventricular samples of the patients appeared to be reduced in the adenosine treated group as reported earlier [28]. However, due to small percentage of apoptotic cells and high variation in the results, this change was not statistically significant. Detection of apoptotic cardiomyocytes is methodologically challenging, because the appearance of DNA fragmentation is a late feature of the apoptotic process and, therefore, TUNEL method may detect only small fraction of eventually apoptotic cells when the samples are taken very shortly after reperfusion [29]. Therefore, we hypothesized that measurement of changes in apoptosis-regulating genes could serve as an early marker of the severity of ischemic myocardial injury as well as provide means to study effects of cardioprotective interventions in tissue biopsies obtained in the early reperfusion period. Although there was a difference between the control and the adenosine treated groups, there are several doubts in this approach. First, the variation between individual patients in the baseline and postoperative expression was high since this is a clinical study and there is always something cannot be controlled in small powered trial. Second, we could not demonstrate relationships either between gene expression and the amount of apoptosis or the amount TUNEL positive cardiomyocytes. Third, the results need to be interpreted cautiously due to the complex interplay between pro- and antiapoptotic regulators.

Therefore, further methodological validation and development is still needed. Future studies should explore at least the use of other genes as potential markers as well as optimal timing of tissue sampling. 

In the overall picture, as expressed in Table 2, the present study has the value to address the facts that CPB (or aortic cross-clamping) has a significant effect on myocardial injury due to the significant amount of apoptotic cells detected after weaning from the bypass. Fast induction of myocardial arrest could be obtained by intraaortic injection of adenosine. This injection was able to modulate the expression of apoptotic genes decreasing the occurrence of apoptosis significantly. 

Some previous studies which were focusing on the effect of fast adenosine induction of myocardial arrest showed the postoperative clinical improvement in this group of patients [20]. As we reported before [24], we could not demonstrate this clinical effect in our setting but this finding does not exclude the effect of adenosine to decrease apoptosis in such patients. Such effect cannot be overlooked during the trials to improve the quality of myocardial protection. These results should be confirmed in more highly powered studies, possibly with more critical patient cohort. Changing the timing of adenosine administration during reperfusion should be considered for further evaluation. 

In conclusion, cardioplegic arrest during CABG surgery results in changes in apoptosis-regulating genes. The change is detectable in myocardial tissue samples obtained very early after reperfusion and therefore, this method may allow assessment of the quality of myocardial protection.

## Figures and Tables

**Table 1:** Base line Patient Characteristics and operative data.

	Adenosine (*n* = 20)	Control (*n* = 20)
Age (years)*	63.4 ± 6.8	65.5 ± 7.3
Gender (male/female)	19/1	17/3
New York Hear Association class III	20	20
EF*	61 ± 5.8%	63 ± 9.7%
Coronary vessels^†^		
LMA	3	3
50–74%		
≥75%	5	3
LAD		
50–74%	7	5
≥75%	11	10
CX		
50–74%	4	3
≥75%	6	8
RCA		
50–74%	5	1
≥75%	8	8
No of Grafted	3	3
vessels*		
CKMB*	3	3
CBP time	94 ± 25	91 ± 16
Ischemic time	79 ± 26	72 ± 18
Weaning time	15 ± 6.4	19 ± 8

*Figures present the mean value, CKMB indicates preoperative value the day before the operation.

^†^Vessels classified regarding to the site of lesion and percentage of stenosis and according to the most recent coronary angiography, which is used to make the decision to operate the patient. Figures present the number of vessels affected in each category.

**Table 2:** TUNEL positive and negative subgroups (of the ventricular samples) in both the adenosine and control groups in correlation with the changes of apoptosis-regulating genes in the atria samples.

		bax		bcl-2		bax/bcl-2	
Group	Subgroup	Before	After	Before	After	Before	After
	TUNEL negative	0.47 ± 0.83	0.53 ± 1.17	0.1 ± 0.07	0.15 ± 0.7	4.97 ± 8.5	3.88 ± 5.6
Adenosine	TUNEL positive	0.91 ± 3.3	0.35 ± 2.4	0.29 ± 1.7	0.08 ± 1.3	2.4 ± 2.4	5.1 ± 5.1
	Total group	0.52 ± 16.5	0.59 ± 2.36	0.104 ± 0.91	0.107 ± 0.79	8.8 ± 13.67	5.1 ± 15.75

	TUNEL negative	1.4 ± 20.5	0.44 ± 1.2	0.28 ± 2.5	0.09 ± 0.3	6.02 ± 5.3	4.38 ± 13.5
Control	TUNEL positive	0.92 ± 4.5	0.64 ± 0.9	0.23 ± 0.5	0.12 ± 0.2	5.21 ± 5.2	7.57 ± 7.5
	Total group	1.37 ± 13.07	0.47 ± 1.04	0.14 ± 1.64	0.08 ± 0.25	6.6 ± 15.8	6.4 ± 20.9
